# Association of preoperative cerebral oxygenation with concurrent neurobehavioral scores in term neonates with congenital heart disease compared to healthy controls

**DOI:** 10.3389/fped.2025.1482257

**Published:** 2025-02-14

**Authors:** Nhu N. Tran, Anna Miner, Eniola Adeleke, Rene Phan, Ken M. Brady, Mary-Lynn Brecht, Philippe Friedlich, Geena Zhou, Vidya Rajagopalan, Bradley S. Peterson, Jodie K. Votava-Smith

**Affiliations:** ^1^ Institute for the Developing Mind, The Saban Research Institute, Children’s Hospital Los Angeles, Los Angeles, CA, United States; ^2^ Fetal and Neonatal Institute, Division of Neonatology, Children’s Hospital Los Angeles, Los Angeles, CA, United States; ^3^ Department of Pediatrics, Keck School of Medicine, University of Southern California, Los Angeles, CA, United States; ^4^ Keck School of Medicine, University of Southern California, Los Angeles, CA, United States; ^5^ Dornsife College of Letters, Arts and Sciences, University of Southern California, Los Angeles, CA, United States; ^6^ Lurie Children’s Hospital of Chicago, Northwestern University Feinberg School of Medicine, Chicago, IL, United States; ^7^ School of Nursing, University of California, Los Angeles, CA, United States; ^8^ Division of Cardiology, Department of Pediatrics, Children’s Hospital Los Angeles, Los Angeles, CA, United States; ^9^ Department of Psychiatry, Keck School of Medicine, University of Southern California, Los Angeles, CA, United States

**Keywords:** cerebral oxygenation, neurobehavior, congenital heart disease, cerebral oxygen extraction, neonates, neurodevelopmental outcome

## Abstract

**Objective:**

1st: To determine the association of cerebral oxygenation (rcSO_2_) and concurrent neurodevelopmental outcomes between neonates with congenital heart disease (CHD) and healthy controls. 2nd: To examine the association of cerebral fractional tissue oxygen extraction (FTOE) with concurrent neurodevelopmental outcomes in the two groups. 3rd: To evaluate how type and severity of CHD influenced the associations in our primary and secondary objectives.

**Study design:**

Our secondary analysis included 137 neonates (74 with CHD and 63 healthy controls). We used linear regression models to examine the association of the predictors (i.e., cerebral oxygenation, FTOE, type and severity of CHD) with the percentage of abnormal neurobehavioral scores (outcome). The models included the main effects of group, rcSO_2_, and a rcSO_2_-by-group interaction (examined differences between groups) with covariates of postconceptional age at exam, sex, ethnicity, and preductal peripheral oxygen saturation on the percentage of abnormal neurobehavioral scores. We also performed separate regression models separately in each group. We used these models for the 2nd and 3rd objectives, replacing rcSO_2_ with FTOE and type and severity of CHD as predictors.

**Results:**

Neonates with CHD had lower rcSO_2_ values (67% vs. 79%; *p* < 0.001) and higher FTOE values (0.27 vs. 0.19; *p* < 0.001) compared to healthy controls. The association of rcSO_2_ with the neurobehavioral scores significantly differed between groups (*p* = 0.004). In the CHD group, increased rcSO_2_ showed a trend toward better neurodevelopmental outcomes. However, increased rcSO_2_ associated significantly with poorer neurodevelopmental outcomes in the healthy group. Additionally, FTOE significantly differed between groups (*p* = 0.012). The CHD group showed a trend towards increased FTOE and poorer neurodevelopmental outcomes. Conversely, increased FTOE associated significantly with better neurodevelopmental outcomes in the healthy group.

**Conclusions:**

The CHD and healthy neonates had significantly different associations of both rcSO_2_ and FTOE with the neurobehavioral scores. Our findings suggest that both increased and decreased rcSO_2_ and FTOE may negatively affect concurrent neurodevelopmental outcomes in neonates. Our findings also imply a critical range of rcSO_2_ values, where extreme oxygenation on either side may be harmful. Neonates with CHD and healthy controls may exhibit different neurodevelopmental responses to increased rcSO_2_ and FTOE due to differing metabolic demands.

## 1 Introduction

Children with congenital heart disease (CHD) are more susceptible to developmental delays (i.e., poorer neurodevelopmental outcomes) compared to their healthy peers (1, 2). Heart defects disrupt blood flow through the heart and affect oxygen delivery to vital organs, such as the brain. Thus, decreased cerebral oxygenation (rcSO_2_), due to a neonate's heart defect, may be a potential mechanism for poorer neurodevelopmental outcomes. Existing studies have identified structural brain changes in CHD infants at the fetal and early neonatal stages, suggesting that differences in cerebral oxygenation and neurodevelopment may occur during the prenatal period (3). Both decreased cerebral oxygenation and increased cerebral oxygen demand can heighten cerebral fractional tissue oxygen extraction (FTOE) and subsequently affect neurodevelopmental outcomes in neonates with CHD (4, 5). Additionally, severity of CHD may contribute to poorer neurodevelopmental outcomes (4).

Previous studies demonstrated that decreased cerebral oxygenation during and after heart surgery associated with poorer future neurodevelopmental outcomes in neonates with CHD (5, 6). Investigators found that neonates with CHD that had lower mean cerebral tissue oxygenation (48%) after cardiac surgery had poorer neurodevelopmental outcomes at 6, 15, and 21 months of age compared to neonates with CHD with higher mean cerebral oxygenation (58%) (5). Similarly, another study found that more time spent with cerebral oxygenation <40% during cardiac surgery associated with poorer motor development in neonates with CHD at ∼five days after surgery (6). However, the association of preoperative cerebral oxygenation with concurrent neurodevelopmental outcomes in neonates with CHD is unknown.

The current state of the literature also lacks studies examining FTOE with concurrent neurodevelopmental outcomes in neonates with CHD. Studies have shown that increased FTOE values associated with poorer neurodevelopmental outcomes in other vulnerable neonate populations, such as premature neonates (7, 8). Increased FTOE (0.24–0.41) during the first days of life associated with poorer motor outcomes at 2–3 years of age in neonates born prematurely (8). A similar study found that preterm neonates with cognitive impairments at 2 years of age had higher FTOE values 15 min after birth compared to those without impairments (6). Although these reports are in the preterm neonatal populations, FTOE may affect neurodevelopmental outcomes similarly in neonates with CHD.

The anatomy and physiology of different cardiac defects can impact perfusion and oxygenation to the brain, and thus neurodevelopmental outcomes. Investigators found that children with single ventricle CHD experienced an increased risk for poorer neurodevelopmental outcomes compared to children with other CHD defects (5, 9, 10). Similarly, infants and toddlers between 6 and 30 months of age with cyanotic CHD were at an increased risk for poorer neurodevelopmental outcomes compared to children with acyanotic CHD (11–13). Despite these findings, no studies have examined the association of cerebral oxygenation with neurodevelopmental outcomes between type and severity of CHD.

Thus, the primary objective of this study was to determine the association of preoperative cerebral oxygenation with concurrent neurodevelopmental outcomes between neonates with CHD and healthy controls. Our secondary objective was to evaluate the association of FTOE with concurrent neurodevelopmental outcomes between the two groups. Our tertiary objectives analyzed how type and severity of CHD (i.e., single ventricle status and cyanotic status) affected the association of cerebral oxygenation and FTOE with neurodevelopmental outcomes. We hypothesized that the association of cerebral oxygenation and FTOE with neurodevelopmental outcomes would differ between the CHD and healthy control groups. We also hypothesized that decreased cerebral oxygenation and increased FTOE would associate with poorer neurodevelopmental outcomes in neonates with CHD. Lastly, we hypothesized that more severe types of CHD would associate with poorer neurodevelopmental outcomes.

## 2 Materials and methods

### 2.1 Study design

We performed a secondary analysis of an ongoing longitudinal observational cohort study being conducted at the Saban Research Institute of Children's Hospital Los Angeles (CHLA) (14–18). We obtained written informed consent from parents of neonates prior to the start of the study of procedures. All procedures related to this study aligned with the Helsinki Declaration of 1975, as revised in 2008, and the national guidelines for human experimentation (Good Clinical Practice). In addition, the Committee on Clinical Investigations of CHLA (CCI #10-00235 & 09-00055) and the AltaMed ethics committee approved this study.

### 2.2 Setting

We recruited neonates with CHD from the intensive care units and fetal cardiology clinics at CHLA. We recruited healthy controls from the well-baby clinic at AltaMed within CHLA and clinics around Los Angeles. We collected data between July 2016 and June 2023.

### 2.3 Participants

We included neonates with CHD if they met the following criteria: (1) ≥37 weeks gestational age at birth, (2) postnatal age ≤14 days, (3) examined between July 2016 and June 2023, (4) clinically stable (please refer to the exclusion criteria below), (5) documented structural heart defect requiring admission to CHLA, and (6) had not yet undergone surgery for the cardiac defect. We included healthy controls if they met criteria 1–4. We excluded neonates if they had documentation of any of the following conditions: (1) pre- or post-natal medical conditions besides CHD, (2) genetic syndrome, (3) intubated on mechanical ventilation at time of assessment, (4) inotropic support (>5 mcg/kg/min), (5) documented infection in the medical record, (6) neurologic injury identified by cranial ultrasound before study procedures e.g., intraventricular hemorrhage, (7) infant of substance abusing mother, (8) maternal chorioamnionitis, (9) maternal steroid use in the last trimester, (10) small for gestational age, and (11) unable to obtain neurobehavioral or cerebral oxygenation data.

### 2.4 Main predictor and outcome variables

We used the percentage of abnormal neurobehavioral scores as the primary neurodevelopmental outcome variable, measured with the Einstein Neonatal Neurobehavioral Assessment Scale (ENNAS). Although neonatal neurobehavioral tests, like the ENNAS, may not provide specified delays, they have been shown to identify global differences when compared to controls. We defined our primary independent variable as cerebral oxygenation. Other independent variables for our secondary and tertiary objectives included FTOE, type of CHD, and severity of CHD (refer to the sections below for more detail).

Keywords: cerebral oxygenation, neurobehavior, congenital heart disease, oxygen extraction, neonates, neurodevelopmental outcome.

### 2.5 Data sources/measurement

#### 2.5.1 Demographic and clinical data

The parent in attendance at the study visit completed a questionnaire which included demographic data, medical history, and cardiac information. We reviewed the electronic medical records for neonates with CHD to determine type and severity of CHD.

#### 2.5.2 Data collection protocol

We performed the following physiological data collection procedure if the neonate was asleep or drowsy before the neurobehavioral assessment (using the ENNAS). We used a standardized procedure to collect cerebral oxygenation data: (1) we placed a neonatal cerebral oxygenation sensor on the center of the forehead, that was connected to the INVOS 5100C device (Somanetics, Troy, MI); (2) we placed a preductal peripheral oxygen saturation (SpO_2_) sensor on the neonate's right hand, that was connected to the Philips IntelliVue MP70 monitor; (3) we connected both monitors to the Bernoulli data acquisition system (Cardiopulmonary Corporation, Milford, CT) which aggregated data from both monitors; (4) we collected cerebral oxygenation and SpO_2_ data while the neonate was in a supine position for two minutes, followed by data collection in a sitting position for two minutes; (5) we repeated this procedure twice for a total of three tilts. Thus, we obtained these data for a total of 12 min. However, we performed the neurobehavioral assessment first, if the neonate was awake, to standardize the drowsy or asleep state for the physiological data collection.

#### 2.5.3 Neurobehavioral assessment (ENNAS)

The ENNAS is a valid and reliable tool for assessing neurobehavioral status in both healthy neonates and those with CHD (19–21). This tool has also demonstrated predictive validity for long-term neurodevelopmental outcomes in vulnerable neonatal populations (22). The ENNAS consists of 26 items to assess neurobehavior in neonates <30 days of age. This tool evaluates neonates for motor strength, newborn reflexes, auditory reactions, and visual responses. We defined abnormal scores as any item scoring below the normative line determined by ENNAS developers. We calculated the percentage of abnormal neurobehavioral scores by dividing the number of abnormal items by the total items scored and multiplying this by 100%. We defined better neurodevelopmental outcomes as a lower percentage of abnormal neurobehavioral scores. We used the percentage of abnormal neurobehavioral scores because we could not administer all 26 items on all neonates due to medical restrictions (e.g., intravenous lines, which affected our ability to perform items that required moving the neonate in a circular pattern) or the state of the neonate (e.g., their eyes were closed during the entire exam, which affected our ability to perform two visual testing items). Thus, the total number of items completed and scored varied due to these circumstances.

#### 2.5.4 Cerebral oxygenation and SpO_2_

We measured cerebral oxygenation using near-infrared spectroscopy (NIRS) with an INVOS 5100C device (Somanetics, Troy, MI) using the data collection protocol described above. We first calculated the average of the two-minute supine values. We then calculated the average of two-minute sitting values. Lastly, we calculated an average of these two values (total two-minute supine value and the total two-minute sitting value), which we defined the cerebral oxygenation value for each participant. We measured SpO_2_ using the Philips IntelliVue MP70 monitor and calculated the average SpO_2_ value for each neonate in the same manner described for cerebral oxygenation.

#### 2.5.5 FTOE

We computed an FTOE value for each neonate using their previously calculated cerebral oxygenation and SpO_2_ values using the following equation: $\;\mathrm{FTOE}=\frac{\mathrm{Sp}O_{2}-\mathrm{rcS}O_{2}}{\mathrm{Sp}O_{2}}$

#### 2.5.6 Type and severity of CHD

We defined type of CHD as one of the following categories: (1) two ventricle, (2) two ventricle with arch obstruction, (3) single ventricle, (4) single ventricle with arch obstruction, and (5) dextro-transposition of the great arteries (d-TGA). We identified severity of CHD two ways: single ventricle status and cyanotic status. We classified each neonate's heart defect as (1) single ventricle or two ventricle and (2) cyanotic or acyanotic. We defined cyanotic CHD as cardiac defects which cause intracardiac mixing of deoxygenated blood into the systemic circulation. We considered single ventricle and cyanotic heart defects to be more severe. A single pediatric cardiologist determined these categorizations for each CHD participant (JKV).

#### 2.5.7 Variables in statistical analyses

We treated all quantitative variables (cerebral oxygenation, FTOE, SpO_2_, and the percentage of abnormal neurobehavioral scores) as continuous variables in the analyses. The variables pertaining to type and severity of CHD (i.e., single ventricle status and cyanotic status) were categorical. The covariates in our models included postconceptional age at time of exam, sex, ethnicity, and SpO_2_.

#### 2.5.8 Statistical methods

We conducted statistical analyses using IBM SPSS Statistics 28 (IBM Corp., Armonk, NY, USA). We summarized categorical data as frequencies with percentages and continuous data as means with standard deviations. We determined normality of the percentage of abnormal ENNAS scores with the Shapiro–Wilk test. A primary linear regression model examined the main effects of group and cerebral oxygenation (independent variables) with a cerebral oxygenation-by-group interaction on the percentage of abnormal neurobehavioral scores (dependent variable), with covariates of postconceptional age at time of exam, ethnicity, sex, and SpO_2_. We used a secondary linear regression model to assess the effects of group and FTOE (independent variables) with an FTOE-by-group interaction on the percentage of abnormal neurobehavioral scores (dependent variable), with the same covariates described previously. We ran tertiary linear regression analyses with type of CHD, single ventricle status, and cyanotic status individually as the main effects (independent variables), the percentage of abnormal neurobehavioral scores (dependent variable), and their interactions with cerebral oxygenation and FTOE separately. We then performed separate regression analyses in each group (neonates with CHD and healthy controls) to examine their relationships with the independent and dependent variables listed above for the primary and secondary analyses. Lastly, we conducted regression analyses separately by type and severity of CHD to examine these associations in the subgroups of the CHD categories to complete the third aim. We considered two-sided *p*-values <0.05 significant.

We excluded neonates that were missing percentage of abnormal neurobehavioral scores, cerebral oxygenation, and SpO_2_ data from our analyses. We also performed sensitivity analyses by removing SpO_2_ from our primary and secondary models to examine the stability of the coefficients. We used the results to determine whether the model was vulnerable to the highly correlated relationship of SpO_2_ and cerebral oxygenation.

## 3 Results

### 3.1 Participants

**Figure 1:**
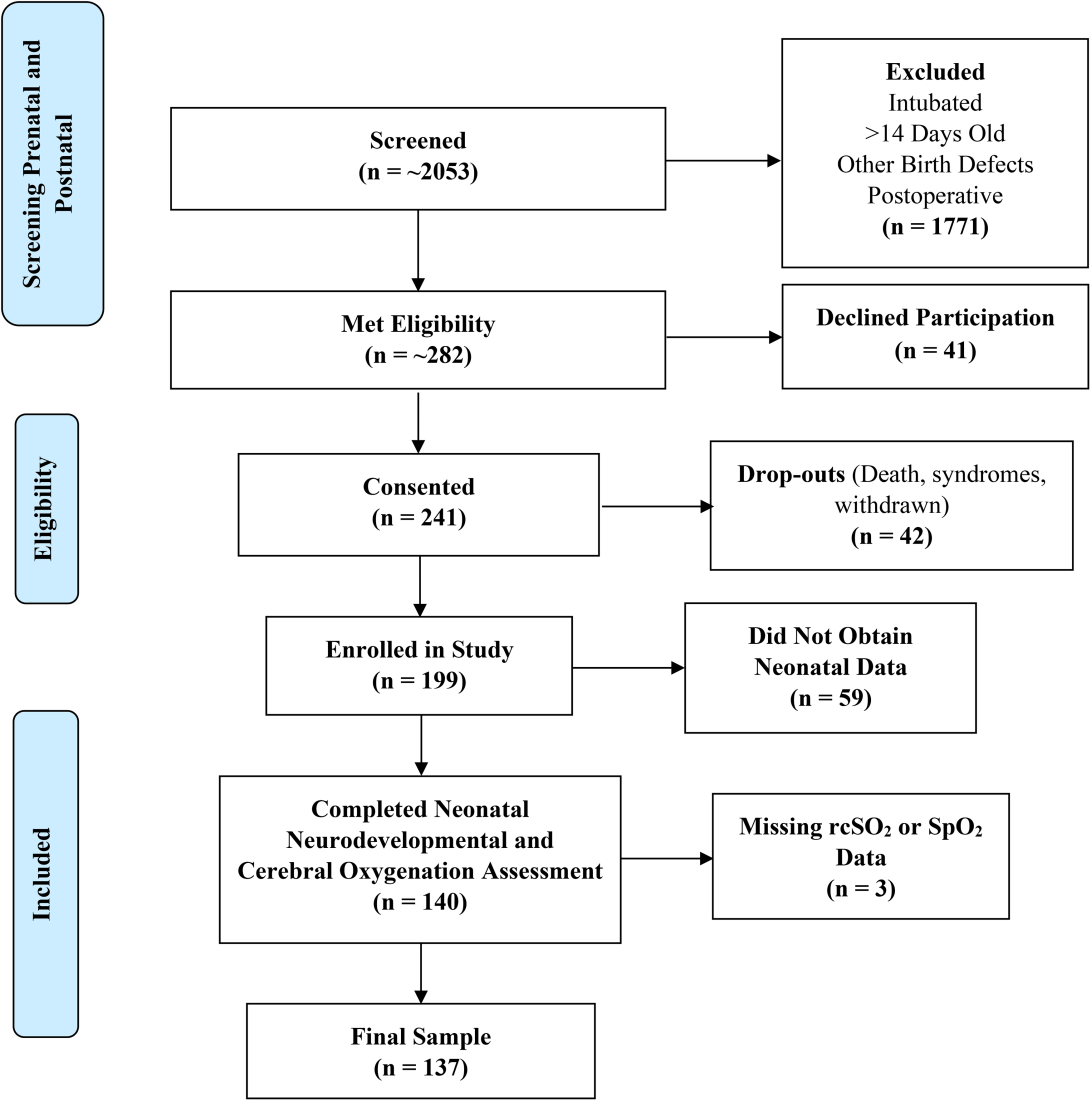
Eligibility flow diagram. rcSO_2,_ regional cerebral oxygen saturation; SpO_2_, preductal peripheral oxygen saturation.

We included 137 participants in the sample (74 neonates with CHD and 63 healthy controls), as seen in Figure 1. Our sample included 48.2% males. Most of our participants were Latino (60.6%), while 16.1% were White, 14.6% were other (African American, mixed, unknown, or other), and 8.8% were Asian/Pacific Islander (Table 1). 65% of the neonates with CHD had two ventricle defects and 69% had cyanotic defects. Table 2 shows a detailed list of the types of heart defects.

**Table 1:** Demographics and physiologic measures of neonates.

	Healthy			CHD			Test statistic	df	*p-*value
	Mean Frequency	± SD	N	Mean Frequency	± SD	N			
Postconceptional age at exam (weeks)	40.22	1.2	63	39.24	1.1	74	5.02	135	<0.001*
Age at exam (days)	8.75	3.34	63	3.55	2.93	74	9.59	125	<0.001*
rcSO_2_ (%)	79.32	6.4	63	66.96	9.6	74	8.94	128	<0.001*
FTOE	0.19		63	0.27	0.08	74	−6.17	135	<0.001*
% Abnormal neurobehavioral scores	0.16	0.09	63	0.31	0.13	74	−7.84	135	<0.001*
SpO_2_ (%)	97.75	1.7	63	91.44	6.6	74	7.96	84	<0.001*
Sex									0.732
Male	46%		29	50%		37			
Female	54%		34	50%		37			
Race/ethnicity							11.4		0.009*
Caucasian	15.9%		10	16.2%		12			
Latino	52.4%		33	67.6%		50			
Asian/Pacific Islander	6.3%		4	10.8%		8			
Other (African American, Mixed, Unknown, Other)	25.4%		16	5.4%		4			

Group comparisons employed either two-sample *t*-tests or chi-square tests/fisher's exact tests. *P*-values were 2-sided.

CHD, congenital heart disease; FTOE, cerebral fractional tissue oxygen extraction; rcSO_2_, regional cerebral oxygen saturation; SpO_2_, preductal peripheral oxygen saturation.

* *p* < .05.

**Table 2:** Subtypes of cardiac defects.

Subtype (*N* = 74)	N (%)
Two ventricle cardiac defects (*N* = 47)	
Aortic stenosis	2 (2.70%)^a^
Arch anomaly with septal defect	11 (14.87%)^a^
AVSD with common atrium	1 (1.35%)
Cortriatriatum sinister	1 (1.35%)^a^
D- TGA	11 (14.87%)
DORV, VSD type with malposed great arteries (d-TGA type) with malposed great arteries (d-TGA type), COA	1 (1.35%)^a^
Isolated arch anomaly	6 (8.11%)^a^
L- TGA With coarctation With VSD	2 (2.70%)^a^
TAPVR	3 (4.05%)
Tetralogy of fallot	6 (8.11%)
Truncus arteriosus	3 (4.05%)
Single ventricle cardiac defects (*N* = 27)	
Hypoplastic left heart syndrome	8 (10.81%)
Single ventricle with pulmonary Stenosis/atresia	13 (17.57%)
Other single ventricle	6 (8.11%)

AVSD, atrioventricular septal defect; DORV, double outlet right ventricle; D-TGA, dextro-transposition of the great arteries; L-TGA, levo-transposition of the great arteries; PA, pulmonary atresia; PS, pulmonary stenosis; TAPVR, total anomalous pulmonary venous return; VSD, ventricular septal defect.

^a^ acyanotic cardiac defects.

### 3.2 Outcome data

Neonates with CHD had: (1) poorer neurodevelopmental outcomes (31% vs. 16% average abnormal neurobehavioral scores, respectively; *p* < 0.001), (2) decreased average cerebral oxygenation values (67% vs. 79%, respectively; *p* < 0.001), and (3) increased average FTOE values (0.27 vs. 0.19, respectively; *p* < 0.001) compared to controls (Table 1). Within the CHD group, neonates with single ventricle CHD had lower average cerebral oxygenation values compared to neonates with two ventricle defects (64% vs. 69%, respectively; *p* = 0.045) and higher average FTOE values (0.29 vs. 0.25, respectively; *p* = 0.049). Similarly, neonates with cyanotic heart defects had lower average cerebral oxygenation values (64% vs. 74%, respectively; *p* < 0.001) and higher average FTOE values (0.29 vs. 0.23, respectively; *p* = 0.006) compared to neonates with acyanotic defects (Table 3). Neonates with d-TGA had significantly lower cerebral oxygenation compared to neonates with two ventricle CHD (58% vs. 68%, respectively; *p* = 0.029) (Table 4, 5). Further detail of the significant differences in physiologic variables between the various CHD types are show in Table 4.

**Table 3:** Physiologic measures by severity of CHD.

	Single ventricle CHD			Two ventricle CHD			Test statistic	df	*p-*value
	Mean	± SD	N	Mean	± SD	N			
rcSO_2_ (%)	64.26	7.26	27	68.50	10.53	47	2.04	69	0.045*
FTOE	0.29	0.08	27	0.25	0.08	47	−2.00	72	0.049*
% of Abnormal neurobehavioral scores	0.32	0.15	27	0.30	0.12	47	−0.71	72	0.478
	Cyanotic CHD			Acyanotic CHD					
	Mean	± SD	N	Mean	± SD	N			
rcSO_2_ (%)	63.70	8.50	51	74.20	8.04	23	5.01	72	<0.001*
FTOE	0.29	0.08	51	0.23	0.08	23	−2.85	72	0.006*
% of Abnormal neurobehavioral scores	0.31	0.14	51	0.31	0.11	23	0.05	72	0.961

Group comparisons employed two-sample *t*-tests. *P*-values were 2-sided.

CHD, congenital heart disease; FTOE, cerebral fractional tissue oxygen extraction; rcSO_2_, regional cerebral oxygen saturation.

* *p* < .05.

**Table 4:** Physiologic measures by type of CHD.

	Two ventricle			Two ventricle with arch obstruction			Single ventricle			Single ventricle with arch obstruction			TGA			Test Statistic	df	*p-*value
	Mean	±SD	N	Mean	±SD	N	Mean	±SD	N	Mean	±SD	N	Mean	±SD	N			
rcSO_2_ (%)	67.79	10.41	16	74.75	7.45	20	62.06	7.75	15	67.01	5.77	12	58.19	6.84	11	9.63	4	<0.001*
FTOE	0.25	0.08	16	0.22	0.07	20	0.30	0.09	15	0.29	0.06	12	0.32	0.07	11	4.24	4	0.004*
% of Abnormal neuro-behavioral scores	0.29	0.14	16	0.32	0.12	20	0.27	0.10	15	0.39	0.17	12	0.30	0.11	11	1.78	4	0.144

Group comparisons employed analysis of variance (ANOVA). *P*-values were 2-sided.

CHD, congenital heart disease; FTOE, cerebral fractional tissue oxygen extraction; rcSO_2_, regional cerebral oxygen saturation.

* *p* < .05.

**Table 5:** Physiologic measures by type of CHD compared to Two ventricle CHD.

	Two ventricle with arch obstruction				Single ventricle				Single ventricle with arch obstruction				TGA			
	Mean Difference	±SE	N	*p*-value	Mean Difference	±SE	N	*p*-value	Mean Difference	±SE	N	*p*-value	Mean Difference	±SE	N	*p*-value
rcSO_2_ (%)	−6.96	2.66	20	0.111	5.73	2.86	15	0.487	0.78	3.03	12	1.000	9.61	3.11	11	0.029*
FTOE	0.03	0.03	20	1.000	−0.05	0.03	15	0.705	−0.03	0.03	12	1.000	−0.07	0.03	11	0.216
% of Abnormal neuro-behavioral scores	−0.03	0.04	20	1.000	0.02	0.05	15	1.000	−0.11	0.05	12	0.339	−0.01	0.05	11	1.000

Group comparisons employed Bonferroni comparison *P*-values were 2-sided.

CHD, congenital heart disease; FTOE, cerebral fractional tissue oxygen extraction; rcSO_2,_ regional cerebral oxygen saturation.

* *p* < .05.

### 3.3 Main results

#### 3.3.1 Cerebral oxygenation and the percentage of abnormal neurobehavioral scores

**Figure 2:**
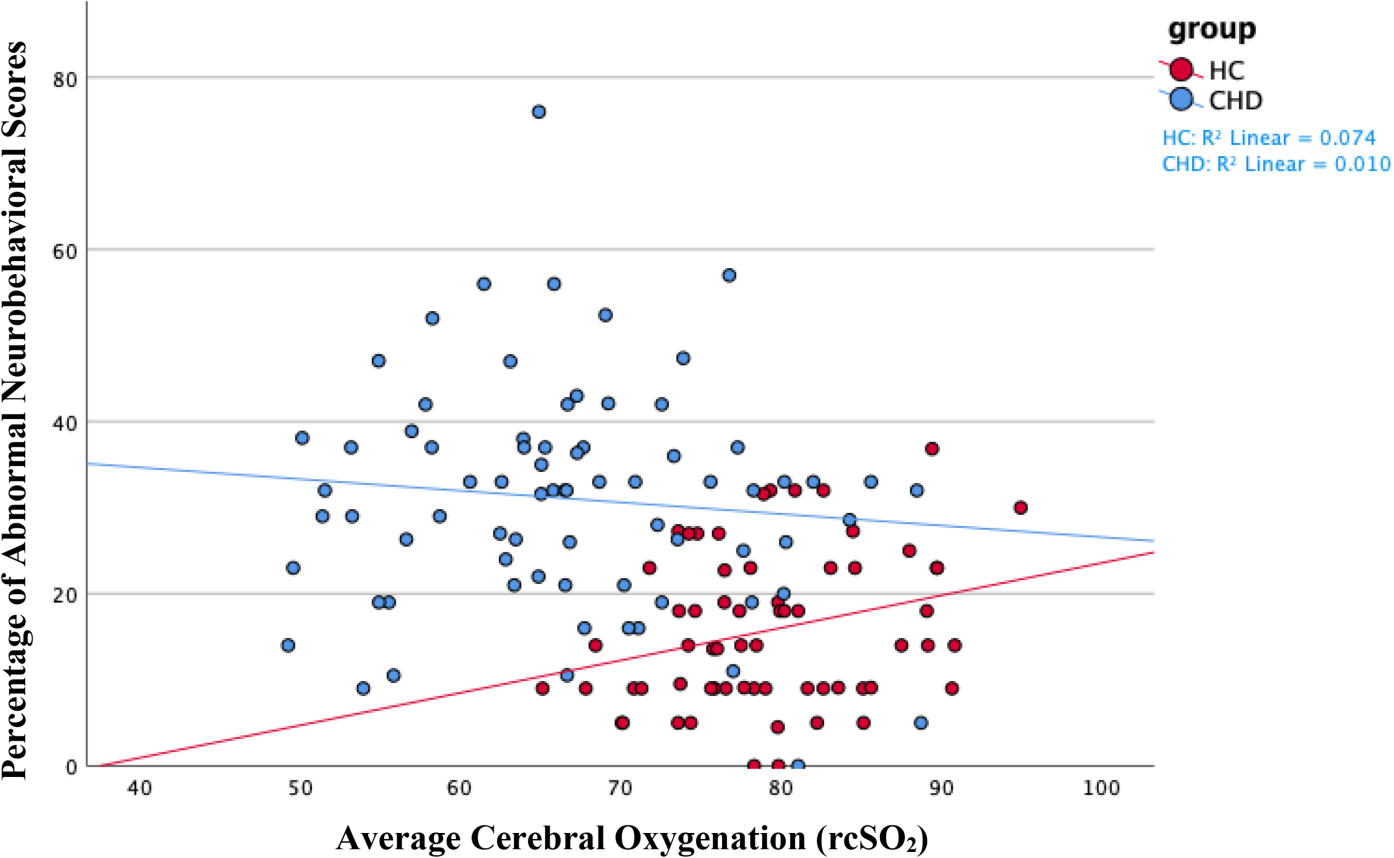
We found a negative association between average cerebral oxygenation and the percentage of abnormal neurobehavioral scores in the entire sample of neonates. In other words, increased cerebral oxygenation associated with a lower percentage of abnormal neurobehavioral scores, indicating better neurodevelopmental outcomes. We found a similar association, although insignificant, within the CHD group, as shown in blue on the scatterplot. The opposite association occurred within the healthy control group—higher cerebral oxygenation associated with poorer neurodevelopmental outcomes—as shown in red. On average, the healthy control group had increased cerebral oxygenation values and better neurodevelopmental scores compared to neonates with CHD. The *Y*-axis shows the percentage of abnormal neurobehavioral scores, which can reach a maximum of 100%, but the highest percentage of abnormal neurobehavioral scores in our sample was less than 80%. Therefore, the *Y*-axis only shows values 0% through 80%. CHD, congenital heart disease; HC, healthy controls; rcSO_2_, regional cerebral oxygen saturation.

The association of cerebral oxygenation with the percentage of abnormal neurobehavioral scores significantly differed between the CHD and the healthy control group [ß=−0.01, 95% CI (−0.01, −0.003), *p* = 0.004] in the sample overall (Table 6 and Figure 2). Within the CHD group, we did not find a significant association of cerebral oxygenation with the percentage of abnormal neurobehavioral scores, but the direction of the effect was towards increased cerebral oxygenation with better neurodevelopmental scores [ß=−0.003, 95% CI (−0.01, 0.001), *p* = 0.102]. In contrast, we found a significant relationship of cerebral oxygenation with the percentage of abnormal neurobehavioral scores within the healthy control group [ß=0.01, 95% CI (0.001, 0.01), *p* = 0.009].

**Table 6:** Association of cerebral oxygenation with poorer neurobehavioral scores in neonates.

Percentage of abnormal neurobehavioral scores	N	*β*	Standard error	95% CI	*p*-value
Postconceptional age at exam (weeks)	137	−0.001	0.01	(−0.17, 0.02)	0.943
Sex	137	0.02	0.02	(−0.01, 0.06)	0.210
Ethnicity	137	0.01	0.01	(−0.01, 0.03)	0.291
Mean SpO_2_ (%)	137	0.01	0.002	(0.001, 0.01)	0.014*
Mean rcSO_2_ (%)	137	0.004	0.002	(0.00, 0.01)	0.053
Group	137	0.80	0.22	(0.37, 1.24)	<0.001*
rcSO_2_-by-Group	137	−0.01	0.003	(−0.14, −0.003)	0.004*

Our primary regression analysis assessed the association of cerebral oxygenation with the percentage of abnormal neurobehavioral scores in the entire sample of neonates. The rcSO_2_-by-Group interaction tested our hypothesis that the relationship between cerebral oxygenation and the percentage of abnormal neurobehavioral scores would differ between neonates with CHD and healthy controls. We found a significant difference in the association of cerebral oxygenation and the percentage of abnormal neurobehavioral scores between groups. Covariates in the model were postconceptional age at time of exam, sex, ethnicity, and SpO_2_.

CHD, congenital heart disease; rcSO_2_, regional cerebral oxygen saturation; SpO_2_, preductal peripheral oxygen saturation.

* *p* < .05.

#### 3.3.2 FTOE and the percentage of abnormal neurobehavioral scores

**Figure 3:**
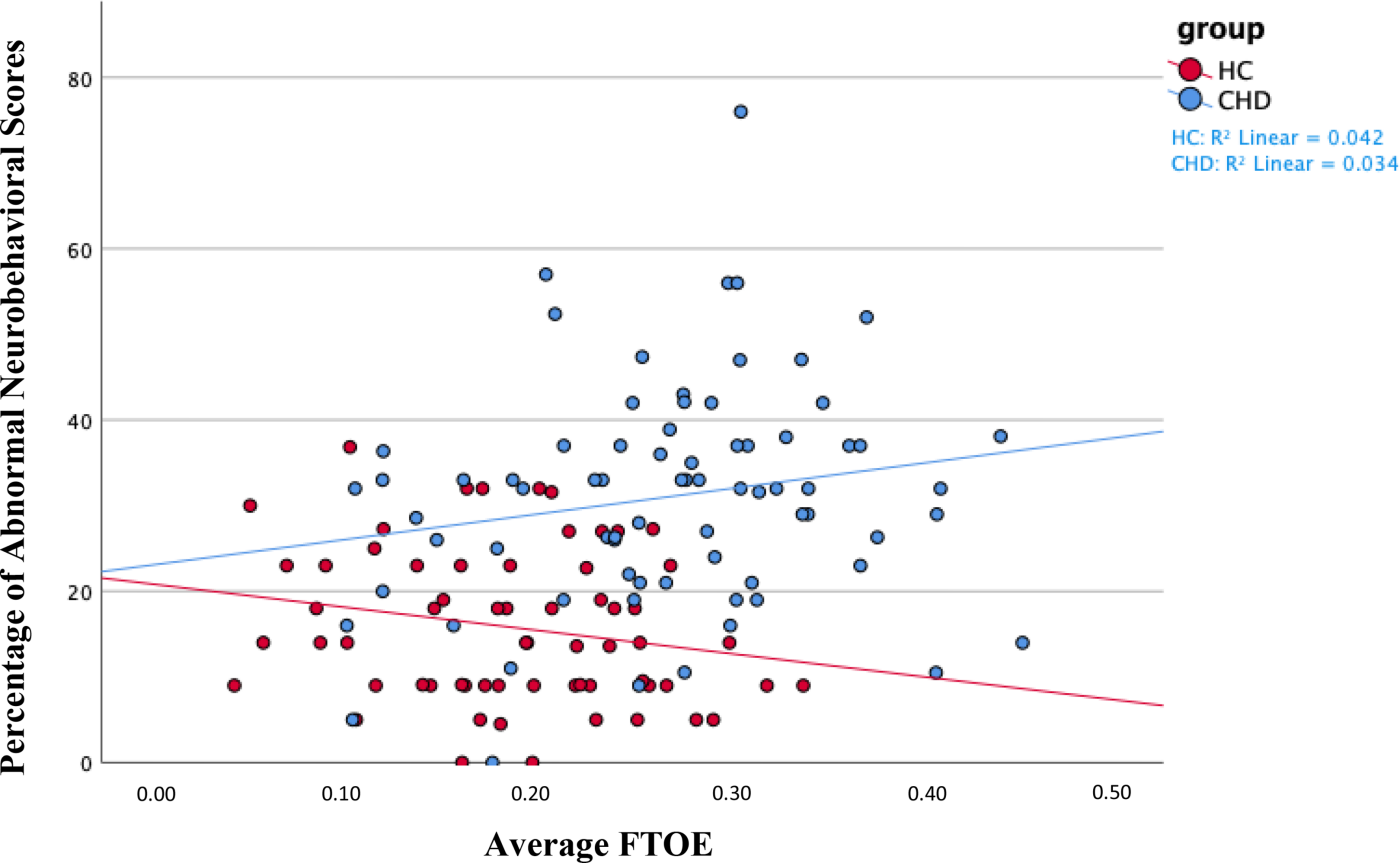
We found a positive association between average FTOE and the percentage of abnormal neurobehavioral scores in the entire sample of neonates. Namely, increased FTOE values associated with a higher percentage of abnormal neurobehavioral scores, indicating poorer neurodevelopmental outcomes. We found the same association, although insignificant, within the CHD group, as shown in blue on the scatterplot. We found the opposite association—increased FTOE associated with better neurodevelopmental outcomes—within the healthy control group, as shown in red. On average, the healthy control sample had decreased FTOE values and better neurodevelopmental outcomes compared to neonates with CHD. The *Y*-axis shows the percentage of abnormal neurobehavioral scores, which can reach a maximum of 100%, but the highest percentage of abnormal neurobehavioral scores in the sample was less than 80%. Therefore, the *Y*-axis only shows values 0% through 80%. CHD, congenital heart disease; FTOE, cerebral fractional tissue oxygen extraction; HC, healthy controls.

We found a significant difference in the association of FTOE with the percentage of abnormal neurobehavioral scores between the CHD group to the healthy control group [ß=0.69, 95% CI (0.15, 1.23), *p* = 0.012], as seen in Table 7 and Figure 3. We did not observe a significant association of FTOE with the percentage of abnormal neurobehavioral scores in the CHD group, but the direction of the association was towards increased FTOE and poorer neurodevelopmental outcomes [ß=0.31, 95% CI (−0.07, 0.69), *p* = 0.110]. In contrast, increased FTOE significantly associated with better neurobehavioral scores within the healthy control group [ß=−0.46, 95% CI (−0.80, −0.12), *p* = 0.010].

**Table 7:** Association of FTOE with poorer neurobehavioral scores in neonates.

Percentage of abnormal neurobehavioral scores	N	β	Standard error	95% CI	*p*-value
Postconceptional age at exam (weeks)	137	−0.001	0.01	(−0.02, 0.02)	0.901
Sex	137	0.03	0.02	(−0.01, 0.07)	0.174
Ethnicity	137	0.01	0.01	(−0.01, 0.04)	0.262
Mean SpO_2_ (%)	137	0.003	0.002	(0.00, 0.01)	0.081
Mean FTOE	137	−0.34	0.21	(−0.78, 0.07)	0.099
Group	137	0.02	0.01	(−0.10, 0.15)	0.719
FTOE-by-group	137	0.69	0.27	(0.15, 1.23)	0.012*

Our secondary regression analysis assessed the association of FTOE with the percentage of abnormal neurobehavioral scores in the entire sample of neonates. The FTOE-by-Group interaction tested our hypothesis that the relationship between FTOE and the percentage of abnormal neurobehavioral scores would differ between neonates with CHD and healthy controls. We found a significant difference in the association of FTOE and the percentage of abnormal neurobehavioral scores between groups. Covariates in the model were postconceptional age at time of exam, sex, ethnicity, and SpO_2_.

CHD, congenital heart disease; FTOE, cerebral fractional tissue oxygen extraction; SpO_2_, preductal peripheral oxygen saturation.

* *p* < .05.

#### 3.3.3 Type and severity of CHD with the percentage of abnormal neurobehavioral scores

Type of CHD did not significantly affect the association of cerebral oxygenation with the percentage of abnormal neurobehavioral scores [ß*=*0.001, 95% CI (−0.002, 0.003), *p* = 0.654]. Similarly, the association of cerebral oxygenation with the percentage of abnormal neurobehavioral scores did not significantly differ by severity of CHD [*single ventricle status* ß=0.003, 95% CI (−0.01, 0.01), *p* = 0.48; *cyanotic status* ß=−0.001, 95% CI (−0.01, 0.01), *p* = 0.784]. Additionally, we did not find a significant difference in the association of FTOE with the percentage of abnormal neurobehavioral scores by type of CHD [ß=−0.93, 95% CI (−0.42, 0.23), *p* = 0.570] or severity of CHD [*single ventricle status* ß=−0.30, 95% CI (−1.14, 0.54), *p* = 0.480; *cyanotic status* ß=0.47, 95% CI (−0.48, 1.37), *p* = 0.308].

#### 3.3.4 Sensitivity analyses

Our sensitivity analyses (removing SpO_2_ from our primary and secondary models) found similar coefficients in the associations of cerebral oxygenation and FTOE with the percentage of abnormal neurobehavioral scores between groups [*cerebral oxygenation* ß=0.01, 95% CI (−0.01, 0.00), *p* = 0.045; *FTOE* ß=0.61, 95% CI (0.08, 1.14), *p* = 0.025].

## 4 Discussion

### 4.1 Key results

The association of cerebral oxygenation with the percentage of abnormal neurobehavioral scores significantly differed between the CHD and healthy control groups, supporting our hypothesis. However, cerebral oxygenation did not significantly associate with the percentage of abnormal neurobehavioral scores in the CHD group, although the direction of the effect was towards increased cerebral oxygenation and better neurodevelopmental outcomes. Interestingly, the healthy controls demonstrated the opposite effect with increased cerebral oxygenation associating significantly with poorer neurodevelopmental outcomes. Similarly, the association of FTOE with the percentage of abnormal neurobehavioral scores significantly differed between groups. The healthy control group had opposite results compared to the CHD group, where increased FTOE significantly associated with better neurodevelopmental outcomes. In contrast, increased FTOE trended in the direction of poorer neurodevelopmental outcomes in the CHD group.

### 4.2 Limitations

Our study had a few limitations. We recruited neonates with CHD admitted to CHLA or mothers prenatally diagnosed with fetuses with CHD. Patient families were referred to CHLA, as it is not a birthing hospital, so these families may have come from areas with access to a tertiary care center. Thus, this group may not be representative of all neonates with CHD, and thus the generalizability of our results is limited to neonates with critical CHD who have access to tertiary care centers and were not intubated and on high levels of inotropic support (>5 mcg/kg/min). Second, we did not include standard prenatal care or other aspects of prenatal health, which could affect *in utero* development, such as diet and exercise during pregnancy, in our inclusion or exclusion criteria. However, we excluded neonates with some outcomes that could result from inadequate prenatal care, such as infection or substance use during pregnancy. Third, our data could be influenced by attending-dependent and institution-dependent management of neonates with CHD. Nevertheless, preoperative management is consistent at CHLA and abides by standard of care, and therefore likely did not skew our findings. Fourth, the neurobehavioral assessments were completed during various times of day at the discretion of each patient's nurse or the family's availability, but often occurred within one hour before or after a feeding. Fifth, the healthy control group was, on average, older in age at time of our measurements compared to neonates with CHD (8.75 days vs. 3.55 days; *p* < 0.001). Age at time of exam may have affected neurobehavioral assessment scores and the differences in scores between the healthy control and CHD groups. Lastly, our sample included 74 neonates with CHD, which may have limited our power to detect a significant difference between type and severity of CHD in our statistical models. However, we powered our study to detect group differences, not differences between types or severity of CHD.

### 4.3 Interpretation

#### 4.3.1 Cerebral oxygenation and neurobehavioral scores

Our sample of neonates with CHD had an average cerebral oxygenation value approximately 12% lower than the average value for the healthy controls. Our findings align with other studies reporting cerebral oxygenation values in neonates with CHD (58%–68%), which are 13%–20% lower compared to healthy controls (76%–80%) (18, 23, 24).

Our study is novel in its evaluation of cerebral oxygenation and neurodevelopmental outcomes in neonates with CHD during in the pre-operative period. No prior reports have examined preoperative cerebral oxygenation with concurrent neurodevelopmental outcomes in neonates with CHD. Thus, we compared our results with reports during and after open heart surgery. One study found low cerebral oxygenation (defined as <40%) during surgery was linked to poorer neurodevelopmental outcomes in neonates and infants with CHD (mean age = 31.2 days) (6). Heart surgery can cause more frequent decreases in cerebral oxygenation due to aortic cross clamp or deep hypothermic circulatory arrest, and NIRS may assess for neuroprotection in that setting. Consequently, this lower average cerebral oxygenation, from heart surgery, likely puts neonates with CHD at an even greater risk for abnormal neurobehavioral scores. Our study, however, obtained clinical and neurodevelopmental data preoperatively and concurrently on neonates who were <14 days old, which may explain our differing results. Other investigators determined that low (56%–71%) and high (84%–92%) quartiles of cerebral oxygenation one day after birth in premature infants associated with poorer cognitive outcomes at 2–3 years of age, and low quartiles of cerebral oxygenation associated with poorer fine motor outcomes (8). These investigators evaluated longer-term neurodevelopmental outcomes where delays tend to be more evident, whereas we examined concurrent neurodevelopmental outcomes. Although these reports examine cerebral oxygenation with future neurodevelopmental outcomes in other high-risk infant populations, the results help explain our findings in the CHD and healthy control groups. Specifically, in our CHD group we observed decreased cerebral oxygenation with poorer neurodevelopmental outcomes, whereas the healthy controls demonstrated increased cerebral oxygenation with poorer neurodevelopmental outcomes. Our results suggest that decreased cerebral oxygenation affects neonates with CHD differently than healthy control neonates, which could be explained by differences in cerebral oxygen extraction in the two groups, discussed in the respective section below.

Neonates with CHD often have reduced cerebral oxygen supply compared to their healthy counterparts as a result of their anatomy (25, 26). Lack of oxygenation can lead to injury or even death of the cerebral tissue (27). Resources carried via blood, such as oxygen and nutrients, are diverted away from injured or dead tissue and towards areas with healthier tissue. While hypoperfusion and hypoxia are problematic for developing neonates, hyperoxygenation also poses a threat to neonatal development via oxidative stress (28). Healthy neonates are better able to counteract oxidative stress compared to vulnerable populations such as preterm neonates or neonates with CHD. Neonates undergo physiologic oxidative stress in the fetal-to-neonatal transition; however, circumstances such as increased oxygen use in the setting of perinatal resuscitation could cause additional oxidative stress in this transitional period (29). We performed parent survey for the inclusion and exclusion of the healthy controls. Although we do not believe this occurred, parent recall may have been biased and they may not remember whether their neonate was resuscitated at birth. Thus, factors such as oxygen use immediately after birth may have contributed to our findings in the healthy group. Mechanistically, healthy neonates should benefit from increased cerebral oxygenation in the same way as neonates with CHD. Therefore, our findings might be better explained by an unaccounted confounding factor rather than an anatomic or physiologic difference between our study groups (28, 30–32). However, the association between cerebral oxygenation and neurobehavioral outcomes may depend on the proximity of baseline oxygenation levels to critical thresholds rather than simply on increases or decreases in cerebral oxygenation values. Our results show that neonates with CHD have lower baseline cerebral oxygenation values compared to healthy infants. Thus, neonates with CHD are more prone to falling below a critically low threshold of cerebral oxygenation, increasing their risk of hypoxic damage, while healthy infants are more likely to exceed critically high thresholds, leading to hyperoxic damage. Thus, an infant's risk of poor neurobehavioral outcomes may depend on their cerebral oxygenation status relative to critical oxygenation thresholds.

Alternatively, our findings could also suggest that the predictive value of cerebral oxygenation in determining neurobehavioral outcomes may vary based on CHD status. While we did not find a significant association between cerebral oxygenation and neurobehavioral outcomes in the CHD group, cerebral oxygenation still remains a valuable tool in clinical practice. Monitoring cerebral oxygenation provides clinicians with critical, non-invasive insights into cerebral oxygen delivery and perfusion, allowing for timely interventions to optimize outcomes in this vulnerable population. Future studies should investigate how cerebral oxygenation metrics can better equip clinicians to improve neuroprotective strategies for neonates with CHD.

#### 4.3.2 FTOE and neurobehavioral scores

FTOE expresses the difference between systemic and cerebral oxygen saturations as a percentage of the systemic saturation. Various studies have established that vulnerable neonatal populations, such as those with CHD or born prematurely, have increased FTOE values compared to healthy controls (33, 34). Neonates with CHD in our sample had a higher average FTOE compared to healthy controls, which aligns with the findings of other studies in neonates with CHD (0.26–0.34) and healthy controls (0.15–0.23) (24, 33).

Our FTOE findings in the CHD group found higher FTOE with poorer neurodevelopmental outcomes, though it did not reach statistical significance. No prior reports have examined FTOE with concurrent neurodevelopmental outcomes in neonates with CHD. Thus, we compared our results with reports in other vulnerable neonatal populations like preterm infants, because both groups are at a higher risk for poorer neurodevelopmental outcomes. Other investigators found that higher FTOE (ranging from 0.24 to 0.55) associated with poorer cognitive and motor outcomes at 2–3 years of age in preterm neonates (7, 8). While our findings in the CHD group did not reach statistical significance, the direction of our results aligned with these studies. These studies may have found significant associations due to a higher range of average FTOE than our CHD group (0.27), which may indicate that preterm infants extract more oxygen than neonates with CHD, thus making their brains more vulnerable to injury. Moreover, FTOE was compared with future developmental outcomes (e.g., the Bayley Scales of Infant and Toddler Development aka Bayley), whereas our study examined concurrent outcomes using the ENNAS. The Bayley may be a more precise tool to identify delays, compared to the ENNAS, and as such our results did not meet statistical significance. Furthermore, neurodevelopmental delays usually become more apparent as children increase in age, thus delays would be easier to identify.

Our CHD and healthy control groups demonstrated opposite associations of FTOE with neurodevelopmental outcomes, which could imply that the outcomes of increased FTOE depends on the clinical context. Neonates with CHD had a higher average FTOE compared to healthy controls and poorer neurodevelopmental outcomes, whereas increased FTOE in healthy neonates showed better neurodevelopmental outcomes. Neonates with CHD have both increased metabolic demand and poorly oxygenated blood, which drives an increase in FTOE at baseline (35–37). In one study, greater FTOE correlated with loss of cerebral autoregulation in preoperative neonates with CHD (38). These vulnerable neonates may need to extract oxygen at high rates to capitalize on limited resources, and may not be able to further augment cerebral perfusion if already subjected to maximal cerebral vasodilation. Further increases of FTOE above an already maximized baseline may have a deleterious effect, resulting in an inability to meet cerebral metabolic demands. Therefore, the CHD infants with higher FTOE are also those with impaired neonatal neurodevelopmental testing. The contrasting association for healthy neonates can be explained with similar logic. Healthy neonates have a normal cardiac circulation to supply adequate oxygen for a standard metabolic demand, and so they have a lower average FTOE compared to neonates with CHD. Thus, an increase in FTOE, in the setting of readily available resources, such as oxygen and nutrients, could imply that healthy neonates are not overextended beyond vital needs of cerebral oxygenation. Therefore, healthy neonates have the capacity to devote additional oxygen to increased metabolic demands, such as during a developmental exam, leading to improved developmental outcomes.

#### 4.3.3 Type and severity of CHD

##### 4.3.3.1 Cerebral oxygenation and FTOE by type and severity of CHD

Our cerebral oxygenation and FTOE results supported the literature for type and severity of CHD. Neonates with single ventricle defects had lower average cerebral oxygenation compared to those with two ventricle heart defects which is similar to the findings of our previous study (18). The average cerebral oxygenation in neonates with single ventricle CHD was fairly consistent with a study that examined pre-operative cerebral oxygenation in neonates with Hypoplastic Left Heart Syndrome (HLHS) (61%) (39). We also found a significant difference in cerebral oxygenation between neonates with cyanotic and acyanotic CHD, intuitive given that the cerebral saturation depends on systemic saturation and thus is expected to be less in cyanotic neonates, which is also in accordance with a prior study (23).

FTOE was significantly higher in neonates with single ventricle and cyanotic defects compared to those with two ventricle defects and acyanotic defects. One study did not find a significant difference in FTOE between neonates with HLHS and d-TGA (40). The differing results may be due to our sample of two ventricle defects included both cyanotic and acyanotic defects, whereas the single ventricle sample had only cyanotic defects. We could not compare our findings for FTOE in cyanotic and acyanotic defects as the current literature does not draw similar comparisons. Our results suggest that more severe heart defects tend to extract more oxygen compared to less severe defects.

##### 4.3.3.2 Neurodevelopmental outcomes by type and severity of CHD

We did not find significant differences in neurodevelopmental outcomes among type or severity of CHD, which corroborates with some reports (41, 42), but deviates from other studies (9). Several investigators reported that children with single ventricle CHD exhibit more impaired cognitive, language, and motor skills at 14.5 months, 2 years, and 3 years compared to those with two ventricle CHD (43–45). These deficits persist through early school age years, as another study that reported diminished visual-motor coordination, memory, and learning abilities in children with single ventricle defects compared to those with two ventricle defects at 5 years of age (46). Our CHD sample may have been too small to detect significant differences in neurodevelopmental outcomes by type and severity of CHD. Each group by type of CHD, for example, was relatively small compared to the total sample size of our study (*N* = 11–20). Furthermore, we examined neurodevelopmental outcomes in the neonatal period and preoperatively, which eliminated confounders such as prolonged hospital stays and effects related to cardiac surgery, which may influence later neurodevelopmental outcomes. On the other hand, a few studies reported no significant differences in neurodevelopmental outcomes between single and two ventricle CHD (41, 42). One investigator found similar cognitive outcomes between infants with single ventricle vs. two ventricle heart defects at 6–8 months of age (47). Those investigators also found worse motor outcomes in infants with single ventricle defects at 6–8 months; but, those differences disappeared in future visits. Those investigators used the Psychomotor Development Index of Bayley to measure motor outcomes in older infants, whereas we used a broader neurobehavioral tool in neonates (i.e., the ENNAS), which could explain these differences in motor outcomes. We expected to find significant differences in associations with neurodevelopmental outcomes between type and severity of CHD. Cyanotic and single ventricle cardiac defects can lead to impairment in cerebral oxygenation and perfusion, and single ventricle heart defects have been associated with decreased brain growth pre and postnatally (48, 49). However, all the infants in this study had severe forms of CHD necessitating neonatal hospital admission and neonatal cardiac intervention, which may underly lack of differences seen between our CHD groups.

## 5 Conclusion

We found a significant difference in the associations of both cerebral oxygenation and FTOE with the percentage of abnormal neurobehavioral scores between neonates with CHD and healthy controls. Each group showed opposite associations with the neurodevelopmental outcomes, highlighting the complex interplay between CHD physiology and cerebral hemodynamics. Increased cerebral oxygenation associated with better neurodevelopmental outcomes for neonates with CHD, (although not significant). Conversely, increased cerebral oxygenation significantly associated with poorer neurodevelopmental outcomes in healthy controls. Similarly, increased cerebral FTOE associated significantly with better neurodevelopmental outcomes for healthy controls. In contrast, increased cerebral FTOE associated with poorer neurodevelopmental outcomes for neonates with CHD (although not significant). These contrasting relationships with neurodevelopmental outcomes suggest that increased cerebral oxygenation and FTOE may have different effects on neonates with and without CHD. Thus, cerebral oxygenation and FTOE monitoring, as well as neurobehavioral exams, may benefit the clinical course, although we understand the difficulties in obtaining these measurements during the preoperative period when the infant may be critically ill. Additional research investigating the ideal levels for both cerebral oxygenation and FTOE will lead to personalized medical management for vulnerable neonates with CHD.

## Data Availability

All data and materials used to conduct this research will be made available upon request.
